# Application of Multigroup Technology in Non-Small-Cell Lung Cancer with Qi Stagnation and Blood Stasis Syndrome

**DOI:** 10.1155/2021/6522191

**Published:** 2021-07-09

**Authors:** Guan-Jun Ma, Xiang Qian, Zhuo Chen, Sha-Sha Chen, Ai-Qin Zhang

**Affiliations:** ^1^Department of Geriatric Oncology, Affiliated Hangzhou Cancer Hospital, Zhejiang University School of Medicine, 34 Yanguan Lane, Hangzhou 310002, China; ^2^Department of Traditional Chinese Medicine, The Cancer Hospital of the University of Chinese Academy of Sciences (Zhejiang Cancer Hospital), 1 Banshandong Road, Hangzhou, Zhejiang 310022, China; ^3^Institute of Basic Medicine and Cancer (IBMC), Chinese Academy of Sciences, 1 Banshandong Road, Hangzhou, Zhejiang 310022, China; ^4^Department of Traditional Chinese Medicine, Taizhou Cancer Hospital, 50 Zhenxin Road, Wenling, Zhejiang 317502, China

## Abstract

**Objective:**

To explore the basic characteristics of intestinal flora, metabolomics, and proteomics of non-small cell lung cancer (NSCLC) in patients with Qi stagnation and blood stasis syndrome.

**Methods:**

Twelve NSCLC patients with Qi stagnation and blood stasis syndrome were selected for the QZXY group and 15 healthy volunteers were selected for the control group. Fecal samples from the two groups were collected to evaluate intestinal microecology using the 16s rDNA technique. Serum samples were collected to compare the differences in metabolomics and proteomics between the two groups using liquid chromatography-mass spectrometry (LC-MS). Another 34 NSCLC patients with other syndromes were selected for the nQZXY group and their serum samples were collected. Metabolomics differences between the QZXY and nQZXY groups were compared using LC-MS, and four metabolites with the most obvious differences were selected for receiver operation characteristic curve representation. Finally, multigroup results were analyzed using the WGCNA software.

**Results:**

There were two significantly different types of bacteria (*Aerococcaceae* and *Abiotrophia*), 11 different proteins (six upregulated and five downregulated), and 38 different metabolites (nine upregulated, 29 downregulated) between the QZXY and control groups. There was a correlation between differential bacteria, proteins, and metabolites. The conjoint analysis found that the different substances were related to MAPK, PI3K/Akt, Ras signaling pathway, cancer pathways, and cytokine-cytokine receptor interaction. There were four significant differences in metabolites *(Pseudouridine*, *phenlacetyl-C0A*, *L-glutamic*, and *phospho-anandamide*) between the QZXY and nQZXY groups.

**Conclusions:**

NSCLC with Qi stagnation and blood stasis syndrome had specific intestinal flora and protein and metabolites, which were closely related to the occurrence and development of tumors.

## 1. Introduction

Lung cancer ranks first in the incidence and mortality rates of malignant tumors in China. Many studies [1–3] have shown that the application of traditional Chinese medicine (TCM) can control tumor progression, improve quality of life, and prolong survival time during treatment of non-small cell lung cancer (NSCLC). TCM may be a strong therapeutic candidate for tumors. Syndrome differentiation is one of the important principles of TCM targeted at recognizing and dealing with diseases. It is based on a comprehensive analysis of clinical information collected using the main TCM diagnosis procedures: observation, listening, questioning, and pulse analyses. In recent years, research on the standardization, objectification, and quantification of TCM syndromes has become popular.

Systems biology is an emerging discipline based on the development of modern and continuous biological experiment technology. Its research methods include transcriptomics, proteomics, metabolomics, and intestinal microecology. The concept of the system is similar to the holistic concept of TCM. TCM syndromes describe the functional state of the body at a certain stage as part of TCM theory. It is a high level generalization of the physiological and pathological state at a certain stage during the occurrence and evolution of the disease. The syndromes can be classified as instantaneous, dynamic, and integral.

Using systems biology methods to objectively study TCM syndromes is conducive to revealing their essence. The TCM pathological properties of lung cancer are as follows: deficiency in origin and excess in excess, the healthy Qi hurts first, and the evil Qi takes the opportunity to enter the lung. Qi stagnation and blood stasis are important pathogenesis processes of lung cancer [4] and [5], and the prescription of regulating Qi and activating blood has a good curative effect [6]. In this study, 16S rDNA and liquid chromatography-mass spectrometry (LC-MS) technology were used to study the proteomics, metabolomics, and intestinal microecology of NSCLC with Qi stagnation and blood stasis syndrome, aiming to provide a biological basis for the diagnosis of TCM syndromes in NSCLC, and an experimental basis for the future of anticancer treatment using Chinese medicine.

## 2. Materials

### 2.1. General Information

The subjects of this study were 46 patients from the thoracic tumor surgery outpatient department of Zhejiang Cancer Hospital between March 1, 2019, and September 30, 2019, who planned to undergo radical resection of lung cancer without any treatment before enrollment. Twelve patients were identified to have the QZXY syndrome by two vice directors or TCM physicians at the same time. The remaining 34 patients were identified as participants with other syndromes (nine patients had spleen deficiency and dampness stagnation (PXSZ) syndrome, 11 patients had a deficiency of Qi and Yin (QYLX) syndrome, 14 patients had Yin deficiency with internal heat (YXNR) syndrome). In addition, 15 healthy volunteers were included.

This clinical study was approved by the Ethics Committee of Zhejiang Cancer Hospital (Approval no. IRB-2018-219).

### 2.2. Diagnostic Criteria of the Patients

  The patients were confirmed to have NSCLC by histopathology and/or cytopathology [7].
  The patients met the following diagnostic criteria of the QZXY syndrome [8, 9], which included a cough that was not smooth, shortness of breath, chest pain as cone or thorns, dry mouth, constipation, hemoptysis or phlegm that is bloody and dark red, lip dark purple tongue, tongue ecchymosis, thin yellow moss, and pulse string or astringent.

### 2.3. Inclusion Criteria of Patients

#### 2.3.1. NSCLC Patients

  Patients meet the diagnostic criteria
  Patients had no history of other malignancies
  Patient ages ranged from 18 to 75 years
  Patients signed the informed consent form in person

#### 2.3.2. Volunteers

  Volunteers had no history of malignancies or major diseases, such as cardiovascular, cerebrovascular, liver, kidney, and endocrine diseases
  Volunteer ages ranged from 18 to 75 years
  Volunteers signed the informed consent form in person

#### 2.3.3. Exclusion Criteria of Patients

  Patients were mentally ill and incapacitated
  Patients or volunteers had been treated with antibiotics for nearly 3 weeks

## 3. Methods

### 3.1. Intestinal Microecological Analysis

Fecal samples (200 mg each) were mixed with 30 mL of buffer solution. After centrifugation at 1,000 rpm for 5 min, the precipitation was collected and resuspended in 10 mL of buffer solution. Fecal microbial DNA was extracted using the QIAAMP Fast DNA Stool Mini Kit. After extraction, the total DNA was inspected using agarose gel electrophoresis and Thermo Nanodrop 2000 UV microspectrophotometer. The samples were amplified in 16S V3–V4 region. The primer information was as follows: forward primer: CCTACGGGNGGCWGCAG; reverse primer: GACTACHVGGGTATCTAATCC. The diluted genomic DNA was used as a template for PCR using Phanta Max Master Mix (Vazyme) high fidelity enzyme. The reaction conditions of PCR were as follows: predenaturation at 95°C for 3°min; 95°C denaturation for 30 s, 55°C annealing for 30s, 72 extension for 45s, and repeat 25 cycles. The final incubation was carried out at 72°C for 5 min. The PCR reaction system included the following: 25 *μ*L of 2 × Phanta Max Master Mix (Vazyme), 2 *μ*L of each primer (10 *μ*M), and DDH2O added to make up a total of 50 *μ*L. PCR product (1.5 *μ*L) was analyzed using 120 V continuous electrophoresis for 20 min in 2% agarose gel. Subsequent UV imaging was performed using the gel imaging system.

After the quality inspection of the library, Qubit was used to quantify the concentration of the mixed library pool, Mixing was carried out according to the volume requirements of each sample. Finally, the Miseq sequencing program was run. Clean reads with the same sequence were sorted according to their abundance, while singletons were filtered out. USearch was used for clustering at a similarity degree of 0.97. After chimeric filtering of the clustered sequences, OTU for species classification was obtained. Finally, all clean reads were matched to the OTU sequence. Reads that could be matched to the OTU were extracted to obtain the final mapped reads.

### 3.2. Proteomic Analysis

A total of 30 *μ*g of protein extract were mixed with 200 *μ*L of 8 M urea. The mixture was centrifuged at 20°C and 14000 R for 15 min. The concentrate was diluted with 200 *μ*L of 8 M urea in 0.1 M Tris-HCl at pH 8.5 and centrifuged. Precipitates were diluted with 100 *μ*L of 8 M urea in 0.1 M Tris-HCl at pH 8.5 and centrifuged again. The concentrate was digested in a wet chamber at 37°C for 12 h with trypsin (enzymatic to protein ratio: 1 : 100, 50 M MABC) and then centrifuged. The digestive solution was collected and rinsed with 50 *μ*L of 0.5 M sodium chloride. The resulting solution was combined with 10% trifluoroacetic acid, acidified, and then desalted using C18Tips (Pierce, Thermo Science Titi C). Activation, equalization, peptide elution, and elution were performed according to the manufacturer's instructions. TIPS were activated with two rinses in 50% acetonitrile (ACN) and equilibrated with two rinses in 0.1% trifluoroacetic acid (TFA) in water. The peptide-rich solution was slowly aspirated and distributed using TIP for 10 cycles. Two washing desalination processes were performed with 0.1% TFA. The desalinated peptide was eluted with 50 *μ*L of 50% can. The eluted product was dried in a vacuum and then suspended in 30 *μ*L of 0.1% formic acid. The solution containing the desalinized peptide (3 *μ*L) was separated using an EASY-Nano-LC system (Thermo Fisher Scientific) and online nanomobile liquid chromatography. The LC was connected to a 2 cm precolumn with an inner diameter of 100 *μ*m and filled with 5 *μ*m of C18 resin (Thermo Fisher Scientific). After the precolumn reaction, the 75 *μ*m × 15 cm capillary column was filled with 3 *μ*m of C18 resin (Thermo Fisher Scientific). The polypeptides were eluted from the column front and analyzed on the column using step-by-step gradient elution, followed by a search of all the original files against the UniProt human protein sequence database in MaxQuant (version 1.6). Perseus was used to calculate the folding changes of each subgroup. Proteomes with more than twofold changes in the data frame (>2 or < 0.5) were labeled as significantly altered proteins.

### 3.3. Metabolomics Analysis

The serum sample (200 *μ*L) was placed on ice at room temperature. Then, 600 *μ*L of methanol/ethyl alcohol (1 : 1), which had been refrigerated overnight at −20°C, was added, swirled for 30 s, and centrifuged at 14,000 rpm at 4°C for 15 min. The supernatant was placed into a centrifuge tube and dried with a vacuum centrifuge concentrator. After drying, the samples were redissolved in acetonitrile/methanol (80/20) and water mixture (1 : 1), swirled for 60 s, and centrifuged at 14,000 rpm at 4°C for 15 min, and 4 *μ*L of the supernatant was extracted and analyzed using UPLC-Q/TOF-MS. The samples were separated on an ExionLC AD ultra-performance liquid chromatography (UHPLC) ACQUITY UPLC HSS T3 column (100 × 2.1 mm, 1.8 *μ*m) using the following specifications: temperature of sample plate: 4°C; column temperature: 40°C; sample size: 4 *μ*L; mobile phase composition A: acetonitrile (containing 0.1% formic acid), B: water (containing 0.1% formic acid); and flow rate: 0.3 mL/min. The elution gradient was 0–1 min, 95%B; 1–20 min, 95∼1%B; and 20–23 min, 1%B. The samples were separated using UHPLC and analyzed with X-500R mass spectrometer (AB SCIEX). The ions with significant differences were screened using single- and multidimensional statistical analysis (*P* < 0.05, FC > 1.5 and VIP > 1.0) as potential biomarkers.

### 3.4. Combined Analysis of Intestinal Microecology and Metabolomics

Spearman's correlation analysis was performed on the secondary differential metabolites screened using metabolomics and significant differential level bacteria obtained via 16S sequencing analysis in order to identify the relationship between microflora and metabolites. Based on the calculation results, appropriate screening conditions were selected to obtain the final correlation and network diagram.

### 3.5. Combined Proteomics-Metabolomics Analysis

By combining metabolomics and proteomics data using the KEGG pathway analysis, proteins and metabolites that are involved in significant changes in the same biological process (KEGG pathway) can be identified and used to quickly target key genes. This process was divided into the following steps: (1) check whether proteome and metabolome are related through KEGG metabolic pathway; (2) data screening for significant difference data and regulatory relationship; (3) GO and KEGG enrichment analysis; (4) clustering of expression patterns for linked data; and (5) creation of linked data network diagram.

### 3.6. Statistical Method

The intestinal floras with significant differences among different groups were identified using a rank-sum test. The threshold value for significance screening was a *P* value < 0.05. All *p* values were verified using FDR. In the analysis of differential proteins, the differentially expressed protein needed to meet the following conditions: the coincidence difference ratio was 1.2 times (up-down) and *P* value < 0.05. The *t*-test method was limited to the comparison between two groups of samples with biological duplicates. When conducting differential metabolite analysis between two groups of samples, univariate analysis can intuitively show the significance of metabolite changes between two samples, thus helping to screen for potential marker metabolites (usually FC > 1.5 and *P* value < 0.05 as the screening criteria). Spearman's correlation coefficient was used for joint analysis. It is a linear correlation coefficient, which is a statistical quantity used to reflect the degree of linear correlation between two variables. The correlation coefficient is expressed using *r*, where *n* is the sample size. It is the observed value and mean value of the two variables, respectively. *R* describes the degree of linear correlation between two variables. The greater the absolute value of *R*, the stronger the correlation. The correlation analysis of significantly different metabolites between the QZXY and nQZXY groups was carried out using the binary logistic regression stepwise method. A receiver operating characteristic curve was used to evaluate the efficacy of differential metabolites in differentiating the two.

## 4. Results

### 4.1. Baseline Subject Data

Twelve NSCLC patients with QZXY syndrome were included in the QZXY group. A total of 34 NSCLC patients with other syndromes (PXSZ, QYLX, and YXNR) were included in the nQZXY group. Fifteen healthy volunteers formed the control group. The above information is presented in Table 1.

### 4.2. Differences between Microecological Groups

#### 4.2.1. Venn Diagram

**Figure 1:**
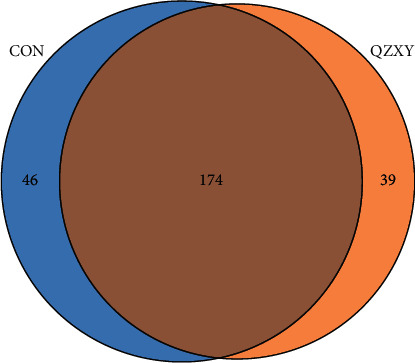
There were 174 common OTUs in the two groups. Compared to the intestinal flora in the control group, significant differences were present in the QZXY group. There were 39 unique OTUs in the QZXY group and 46 unique OTUs in the control group.

The 16 S sequencing analysis showed that a total of 249 OTUs were generated from 27 samples (Figure 1).

#### 4.2.2. Differential Species Analysis

**Figure 2:**
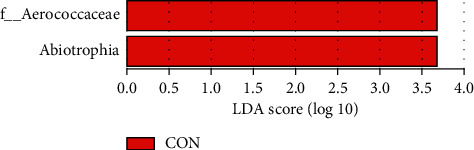
The bacterial genera with significant differences between the two groups (*P* < 0.05) were Aerococcaceae and Abiotrophia. The abundance level in the QZXY group was significantly lower than that in the control group.

Linear discriminant analysis (LDA) effect size (LEFSE) analysis used linear discriminant analysis (LDA) to estimate the influence of the abundance of each component (species) on the difference effect and to determine the communities or species that had a significantly different influence on sample division. The LDA scores obtained using linear regression analysis for the significant microbial groups are shown in Figure 2.

#### 4.2.3. Analysis of Spearman's Correlation Coefficient in Different Species

**Figure 3:**
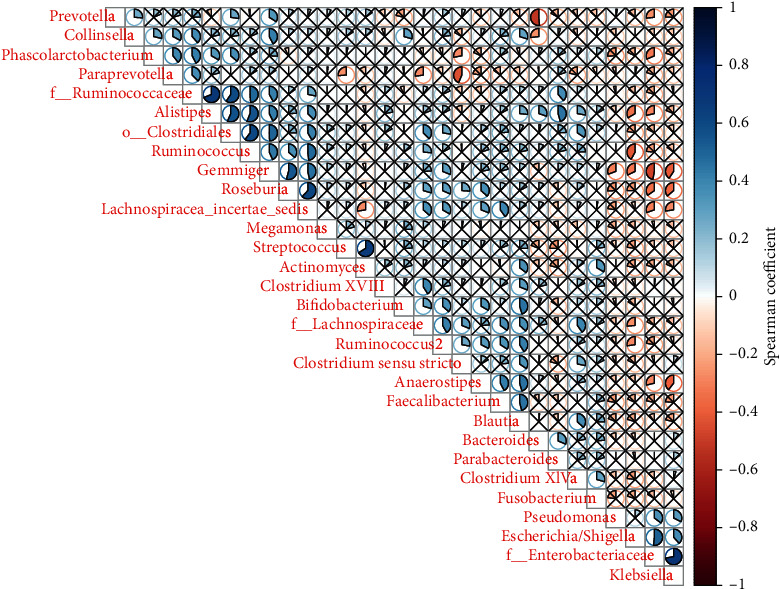
Spearman's correlation coefficient analysis of different species showed that there was a positive correlation between Roseburia and Lachnospiraceae incertae sedis. There was a positive correlation between *Streptococcus* and Actinomyces and between Enterobacteriaceae and *Klebsiella*.

LEFSE was used at each level or the rank-sum test was used at the genus level (or at a specific level) to select the species with different abundance TOP30. Spearman's correlation heat map for species was created using the Corrplot package of *R* software. The specific results are shown in Figure 3.

### 4.3. Differences between Proteomics Groups

#### 4.3.1. Statistical Analysis of Upregulation and Downregulation of Differential Proteins

**Figure 4:**
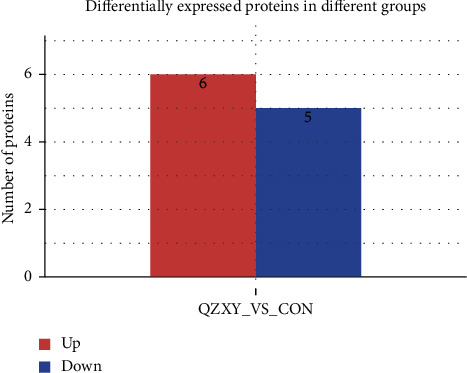
Compared to the control group, there were six upregulated proteins and five downregulated proteins in the QZXY group, for a total of 11 differential proteins.

The up- and downregulation frequency statistics for differential proteins were used to determine the number of differential proteins under different experimental conditions. The horizontal axis represents the comparison group information, and the vertical axis represents the number of proteins. Red identifies the upregulated proteins, blue shows the downregulated proteins, and numbers represent the up- and downregulated proteins (Figure 4).

#### 4.3.2. Functional Analysis of Differential Protein Genes

**Figure 5:**
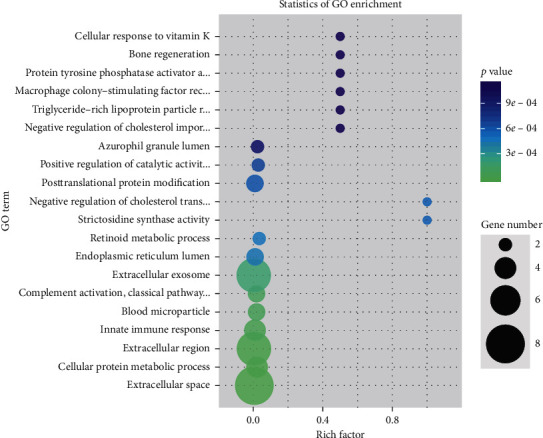
GO results showed that the proteomic genes in the QZXY group are extracellular space and extracellular exosome and can enhance the ability of metastasis and invasion of lung cancer cells and mediate tumor immunosuppression.

The term is the basic unit of GO analysis, and each term corresponds to an attribute. The GO significance enrichment analysis first mapped all of the differentially expressed genes to each term in the GO database, calculated the number of genes in each term, and then applied a hypergeometric test to identify the GO terms significantly enriched in the differentially expressed genes compared to the entire genome background. The results are shown in Figure 5.

#### 4.3.3. KEGG Pathway Analysis of Differential Protein

**Figure 6:**
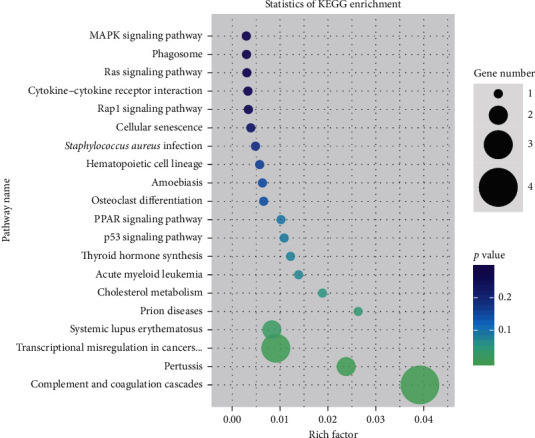
Pathways most associated with outcomes in the QZXY group included MAPK signaling pathway, Phagosome, Ras signaling pathway, and cytokine-cytokine receptor interaction. Tumor-related pathways were also analyzed.

The KEGG pathway was used as a unit of pathway enrichment analysis. The hypergeometric test was used to identify the pathways that were significantly enriched in the differentially expressed genes compared to the overall genome background. The results are shown in Figure 6.

### 4.4. Differences between Metabolomics Groups

**Figure 7:**
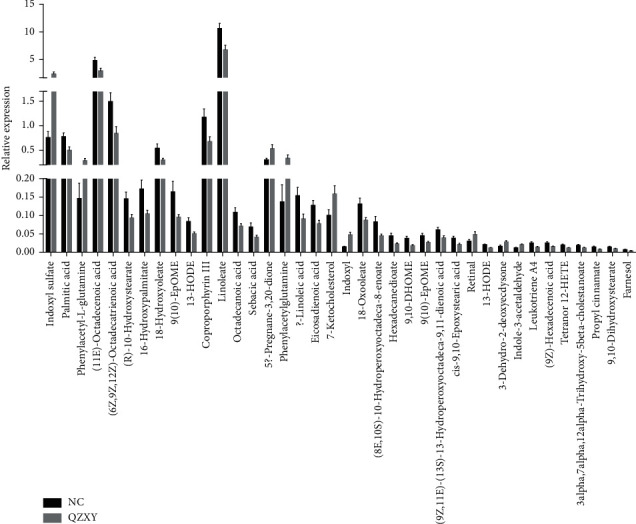
Comparison of different metabolite intensities and specific degrees of differences between QZXY and control groups.

In this study, univariate statistical analysis and multivariate statistical analysis were used to screen for significant difference metabolites. MetDNA and OSI/SMMS software were used for the structure identification of metabolites with significant differences. The specific identification results are shown in Table 2 and Figure 7.

The obtained differential metabolites were submitted to the Metaboanalyst 4.0 website for metabolic pathway analysis. The results are shown in Table 3.

### 4.5. Combined Analysis of Intestinal Microecology and Metabolomics

#### 4.5.1. Correlation Analysis of Differential Metabolites and Differential Flora

**Figure 8:**
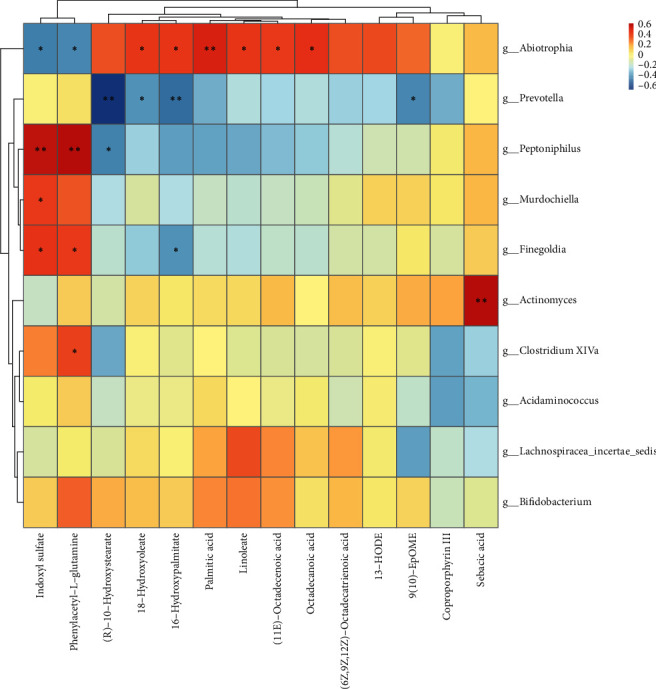
Correlation between anion metabolites and differential flora.

**Figure 9:**
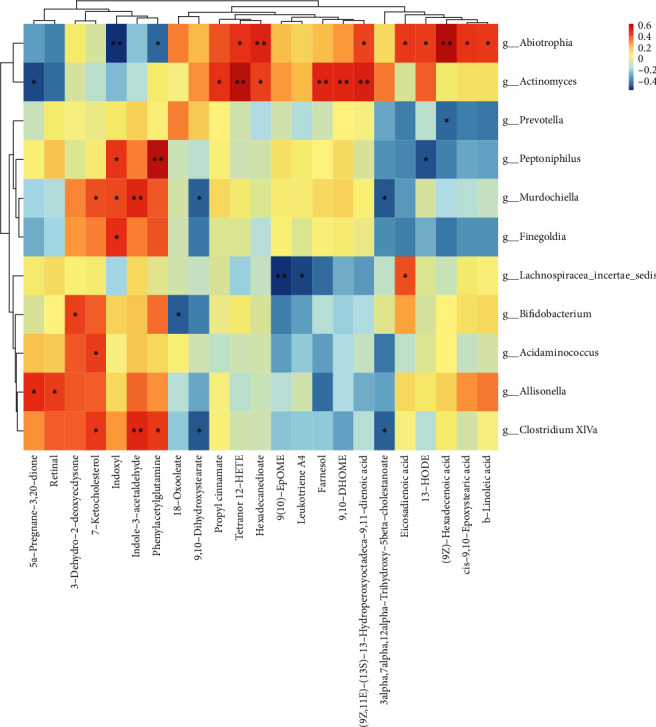
Correlation between positive ion metabolites and differential flora.

The combined analysis results of microflora and metabolites are shown in Figures 8 and 9, where red identifies a positive correlation and blue signifies a negative correlation.

Figures 8 and 9 show that Peptoniphilus was positively correlated with phenylacetyl-L-glutamine and indoxyl sulfate. Prevotella was negatively correlated with phenylacetyl-L-glutamine and 16-hydroxypalmitate. Bifidobacteria and 18-hydroxyoleate were also negatively correlated.

#### 4.5.2. Differential Metabolites and Microflora Network Regulation Analysis

**Figure 10:**
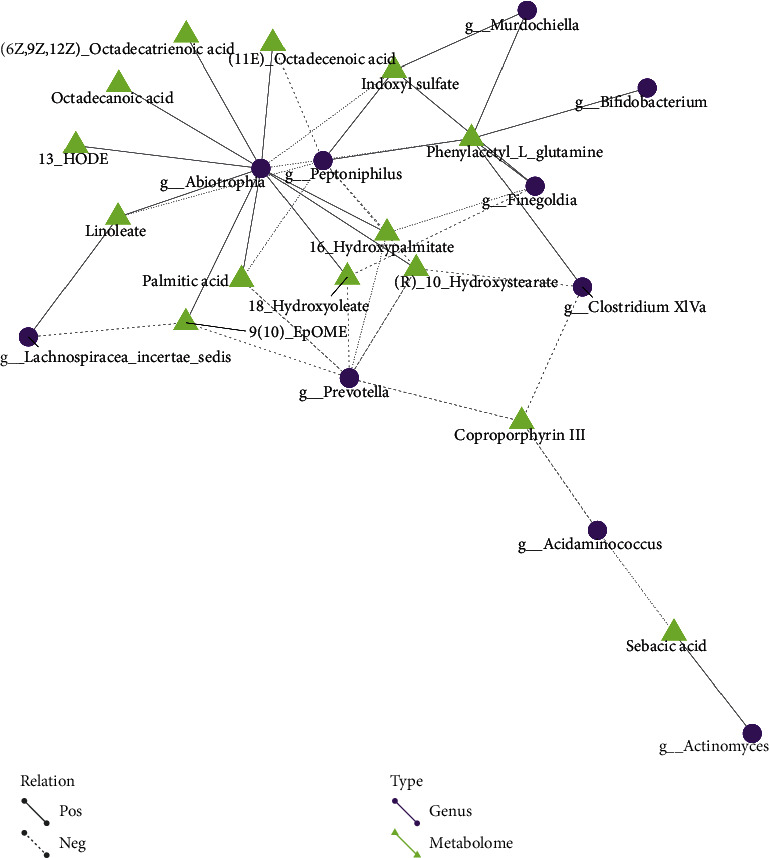
Results of differential metabolite and flora network regulation analysis in anion mode show that Abiotrophia and Peptoniphilus are closely related to many metabolites. This suggests that they may be the key bacteria in the development of lung cancer.

**Figure 11:**
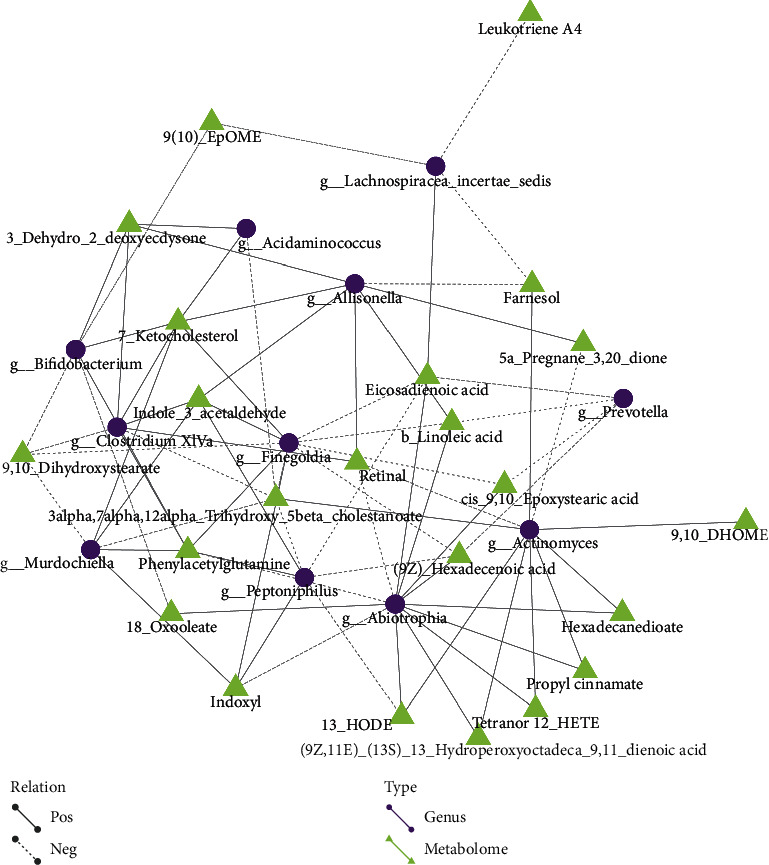
Results of differential metabolite and flora network regulation analysis in positive mode show that *Clostridium XIVa*, *Aclinomyces*, *Finegoldia*, *Abiotrophia*, and *Peptoniphilus* are closely related to many metabolites. This suggests that they also may be the key bacteria in the development of lung cancer.

Different nodes in the figure represent different flora (round) or metabolites (triangular). The line between the flora and the metabolites represents the correlation between the two, where a solid line identifies a positive correlation and a dashed line represents a negative correlation. The results are shown in Figures 10 and 11.

#### 4.5.3. Combined Proteomics-Metabolomics Analysis

**Figure 12:**
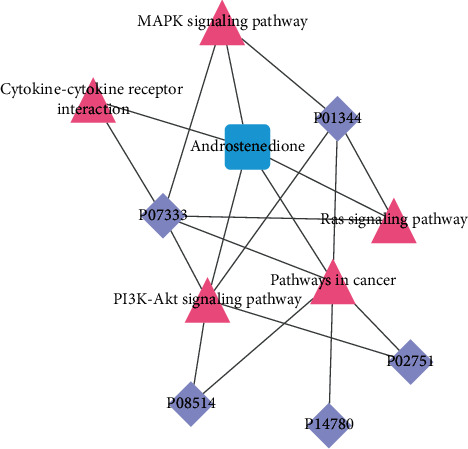
The patient-based differential proteins IGF2, CSF1R, ITA2B, MMP9, FINC, and the downstream metabolite androstenedione were associated with MAPK, PI3K/Akt, Ras signaling pathway, while cancer pathways and cytokine-cytokine receptor interactions were correlated.

The results of the combined proteomics-metabolite analysis are shown in Figure 12.

#### 4.5.4. Metabolite Analysis of Difference between the QZXY Group and the nQZXY Group

**Figure 13:**
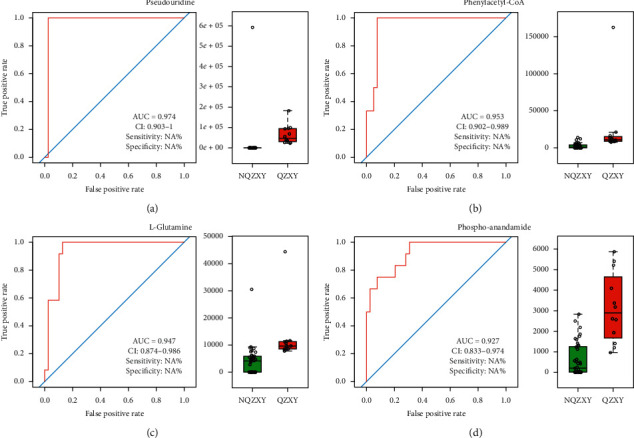
Pseudouridine, phenlacetyl-CoA, L-glutamine, and phospho-anandamide had the highest area under the curve for identification of the two, where their sensitivity was 97.4%, 92.3%, 98.9%, and 92.3% and specificity was 100%, 100%, 100%, and 75%, respectively. The AUC values for other metabolites were all <0.9. (a) Pseudoridine. (b) Phenylacetyl-CoA. (c) L-Glutamine. (d) Phospho-anandamide.

ROC curves were used to analyze the different metabolites between the two groups. The results are shown in Figure 13.

## 5. Discussion

At present, the NSCLC treatment mainly involves different comprehensive treatment strategies according to pathological tumor types and clinical stages, which can somewhat improve the clinical symptoms and prolong survival time. However, individual differences lead to great variations in prognosis [10]. Increasing evidence [11, 12] has indicated that TCM with syndrome differentiation can improve the current situation. However, its validity has always been questioned because of the undefined molecular mechanism of syndromes. The present report attempted to provide a scientific explanation for the syndrome by observing the biological basis for the syndrome's type of Qi stagnation and blood stasis in NSCLC patients.

The present study found that peptone bacteria were positively correlated with glutamine, indolophenol, and other metabolites through network regulation analysis of differential metabolites and flora. The upregulated metabolite glutamine was found to inhibit the migration of human lung adenocarcinoma A549 cells, possibly by downregulating the expression of TNF-*α* and NF-ΚB p65 protein [13]. *Indophenol* can be produced by isopentenylation of tryptophan derivatives. Tryptophan has been found to be metabolized rapidly in many tumors. *Peptoneophilus* is positively correlated with metabolites, such as *glutamine* and *indophenol*. It has been found to be enriched in endometrial carcinoma tissues [14]. No correlation has been found between peptones and lung cancer. This suggests that peptones may affect the formation and development of lung cancer by regulating a variety of metabolic pathways. *Prevotella* and *Bifidobacteria* were negatively correlated with lipid metabolites (palmitic acid, oleate ester). *Palmitic acid* can promote the epithelial-mesenchymal transformation of lung tumor cells and thus enhance their invasion. Microflora has been shown to be involved in host regulation of metabolism and in the production of hormones and bile acids [15]. The present study found that many bacterial genera were negatively correlated with lipid metabolism. This suggested that there may be a certain mechanism between bacterial flora and lipid metabolism to regulate the occurrence and development of lung cancer. NSCLC patients with QZXY syndrome often exhibit blood stasis signs, such as stasis spots on the tip of the tongue. Studies [16] have shown that application of Danshensu, an extract of Salvia miltiorrhiza with the effect of promoting blood circulation and removing blood stasis, can effectively regulate dyslipidemia in hyperlipidemia rats, providing a theoretical basis for the correlation between abnormal lipid metabolism and QZXY syndrome.

The present study explored the changes in signaling pathways in NSCLC patients with QZXY at the multiomics level. Five signaling pathways, including MAPK, PI3K-AKT, Ras, cancer pathways, and cytokine-cytokine receptor interaction, were screened using the linked data network diagram. As a traditional Chinese medicine for promoting blood circulation and removing blood stasis, the active ingredients in Panax notoginseng can act on EGFR, MAPK14, and other targets, regulating cancer pathways, cytokine-cytokine receptor interactions, and other signaling pathways and exert anti-inflammatory effects [17]. It was also found that corydus can exert the effect of promoting blood circulation by regulating the cytokine-cytokine receptor interaction and other signaling pathways [18]. The combination of *Astragalus* and Zedoary (toning Qi and activating blood circulation) can downregulate the expression of MAPK and inhibit the growth and metastasis of transplanted Lewis lung cancer tumors in tumor-bearing mice [19]. To summarize, we speculated that the above five pathways were closely related to lung cancer. Further studies may help to explore how to use TCM theories combined with biomarkers to deeply explore the mechanism of TCM treatment for lung cancer, which is particularly important and can provide new targets for clinical treatment.

We also attempted to analyze the metabolomic differences between the QZXY and nQZXY groups. Four metabolites with ROC > 0.9 and high sensitivity and specificity were found. Pseudouridine is a component of tRNA synthesized by the human body, which can significantly reduce the ability of RNA to stimulate the immune system. It may help lung cancer cells to avoid an immune response. L-Glutamine has also been analyzed. The study results indicated that compared to NSCLC patients with other syndromes, there were significant differences in metabolites in patients with QZXY syndrome. This meant that QZXY syndrome could be diagnosed and differentiated from other syndromes, suggesting that there was microscopic evidence of its presence. However, the sample size in the present study was small, potentially creating a source of bias. Therefore, these results only provided a limited reference value for future investigations. One prior study [20] has found that the syndromic types had different effects on tumor progression by establishing the “Zheng-first” and “Tumor-first“ models of subcutaneous pancreatic cancer in mice with different syndromic types. These results may provide a direction for follow-up studies.

The study of TCM syndromes using systems biology methods is helpful to provide an objective basis for syndrome differentiation and treatment and to suggest highly effective and low-toxicity individualized treatment for tumor patients. In this study, combined multiomics analysis was conducted in NSCLC patients with QZXY. This investigation not only promoted the study of lung cancer mechanisms and determine its pathogenic targets, but also provided theoretical support and technical foundation for the prevention and treatment of lung cancer using traditional Chinese medicine.

## 6. Conclusions

NSCLC with Qi stagnation and blood stasis syndrome had specific intestinal flora and protein and metabolites, which were closely related to the occurrence and development of tumors.

## Figures and Tables

**Table 1:** Sex, age, and stage data for the three groups.

Group	Gender		Average age	Clinical staging		
	Male	Female		I	II	III
QZXY	8	4	59.25 ± 9.60	10	2	0
Control	7	8	61.40 ± 7.02	0	0	0
nQZXY	19	15	58.76 ± 8.67	29	3	2

Statistical analysis indicated that the baseline data were comparable.

**Table 2:** A total of 38 differential metabolites were identified in this study, including 14 metabolites in negative ion mode and 24 metabolites in positive ion mode.

Number	RT (min)	m/*z* (Da)	*P* value	FC	VIP	Adduct	Formula	Compound name
1	4.09	212.0018	0.000752	0.31934	9.74487	M-H	C_8_H_7_NO_4_S	Indoxyl sulfate
2	19.54	255.2324	0.011998	1.545	3.29746	M-H	C_16_H_32_O_2_	Palmitic acid
3	4.08	263.1036	0.029296	0.50604	2.40863	M-H	C_13_H_16_N_2_O_4_	Phenylacetyl-L-glutamine
4	19.72	281.2472	0.018968	1.6259	8.45263	M-H	C_18_H_34_O_2_	(11E)-Octadecenoic acid
5	17.53	277.2166	0.00647	1.7695	5.45746	M-H	C_18_H_30_O_2_	(6Z,9Z,12Z)-Octadecatrienoic acid
6	15.77	299.2594	0.011316	1.5535	1.449	M-H	C_18_H_36_O_3_	(R)-10-Hydroxystearate
7	13.9	271.228	0.008923	1.6475	1.6999	M-H	C_16_H_32_O_3_	16-Hydroxypalmitate
8	14.78	297.2434	0.01004	1.8186	3.22444	M-H	C_18_H_34_O_3_	18-Hydroxyoleate
9	14.48	295.228	0.014379	1.7213	1.66762	M-H	C_18_H_32_O_3_	9(10)-EpOME
10	13.86	295.2284	0.00243	1.658	1.28566	M-H	C_18_H_32_O_3_	13-HODE
11	6.24	653.2632	0.012589	1.7245	4.57453	M-H	C_36_H_38_N_4_O_8_	Coproporphyrin III
12	18.58	279.2312	0.004448	1.5613	13.3247	M-H	C_18_H_32_O_2_	Linoleate
13	20.9	283.2648	0.008692	1.5262	1.27355	M-H	C_18_H_36_O_2_	Octadecanoic acid
14	7.83	201.1134	0.0214	1.6643	1.00239	M-H	C_10_H_18_O_4_	Sebacic acid
15	19.37	317.2474	0.030716	0.58057	4.42563	M + H	C_21_H_32_O_2_	5*α*-Pregnane-3,20-dione
16	4.17	265.1179	0.018487	0.40271	4.31748	M + H	C_13_H_16_N_2_O_4_	Phenylacetylglutamine
17	14.79	281.2474	0.015381	1.6879	2.37695	M + H	C_18_H_32_O_2_	*β*-Linoleic acid
18	19.94	309.2791	0.003259	1.6437	2.33255	M + H	C_20_H_36_O_2_	Eicosadienoic acid
19	20.45	401.3418	0.04793	0.63495	2.25178	M + H	C_27_H_44_O_2_	7-Ketocholesterol
20	4.18	134.0596	0.0004	0.31355	2.05813	M + H	C_8_H_7_NO	Indoxyl
21	21.67	297.2424	0.012856	1.5015	1.91806	M + H	C_18_H_32_O_3_	18-Oxooleate
22	20.72	315.2535	0.013926	1.8311	1.81478	M + H	C_18_H_34_O_4_	(8E,10S)-10-Hydroperoxyoctadeca-8-enoate
23	13.36	287.2217	0.002671	1.9422	1.50515	M + H	C_16_H_30_O_4_	Hexadecanedioate
24	15.11	315.2539	0.001583	2.062	1.47203	M + H	C_18_H_34_O_4_	9,10-DHOME
25	15.53	297.2433	0.002945	1.6887	1.40775	M + H	C_18_H_32_O_3_	9(10)-EpOME
26	14.22	313.2379	0.010082	1.5503	1.36518	M + H	C_18_H_32_O_4_	(9Z,11 E)-(13S)-13-Hydroperoxyoctadeca-9,11-dienoic acid
27	14.79	299.2581	0.005956	1.7695	1.301	M + H	C_18_H_34_O_3_	cis-9,10-Epoxystearic acid
28	19.37	285.2216	0.031826	0.63012	1.23636	M + H	C_20_H_28_O	Retinal
29	13.86	297.2427	0.000278	1.7256	1.05818	M + H	C_18_H_32_O_3_	13-HODE
30	11.96	447.3107	0.022674	0.59386	1.04682	M + H	C_27_H_42_O_5_	3-Dehydro-2-deoxyecdysone
31	6.18	160.0756	0.001919	0.5921	1.04347	M + H	C_10_H_9_NO	Indole-3-acetaldehyde
32	15.53	319.2255	0.007767	1.7745	1.02064	M + H	C_20_H_30_O_3_	Leukotriene A4
33	13.9	255.2319	0.003714	1.6028	0.993851	M + H	C_16_H_30_O_2_	(9Z)-Hexadecenoic acid
34	12.57	267.1955	0.006426	1.7148	0.882565	M + H	C_16_H_26_O_3_	Tetranor 12-HETE
35	17.74	451.3423	0.016417	1.5795	0.807997	M + H	C_27_H_46_O_5_	3alpha,7alpha,12alpha-Trihydroxy-5beta-cholestanoate
36	8.72	191.1065	0.02261	1.7698	0.675408	M + H	C_12_H_14_O_2_	Propyl cinnamate
37	17.86	317.2692	0.016669	1.5145	0.667866	M + H	C_18_H_36_O_4_	9,10-Dihydroxystearate
38	13.36	223.2056	0.001659	1.6801	0.580941	M + H	C_15_H_26_O	Farnesol

**Table 3:** A total of 11 differential metabolic pathways were identified in this study.

Pathway name	Match status	*P*	−log (*p*)	Holm p	FDR	Impact	Details
Linoleic acid metabolism	3/5	3.2013*E* − 5	10.349	0.0026891	0.0026891	1.0	KEGG SMP
Biosynthesis of unsaturated fatty acids	4/36	0.0018721	6.2807	0.15538	0.078626	0.0	KEGG
Retinol metabolism	1/17	0.23408	1.4521	1.0	1.0	0.24227	KEGG SMP
Porphyrin and chlorophyll metabolism	1/30	0.37663	0.97648	1.0	1.0	0.02955	KEGG
Arachidonic acid metabolism	1/36	0.4335	0.83587	1.0	1.0	0.07615	KEGG SMP
Fatty acid elongation	1/39	0.46003	0.77646	1.0	1.0	0.0	KEGG SMP
Fatty acid degradation	1/39	0.46003	0.77646	1.0	1.0	0.0	KEGG SMP
N-Glycan biosynthesis	1/41	0.47705	0.74013	1.0	1.0	0.0	KEGG
Tryptophan metabolism	1/41	0.47705	0.74013	1.0	1.0	0.0139	KEGG SMP
Primary bile acid biosynthesis	1/46	0.51739	0.65896	1.0	1.0	0.03267	KEGG SMP
Fatty acid biosynthesis	1/47	0.52509	0.64419	1.0	1.0	0.01473	KEGG SMP

## Data Availability

The data used to support the findings of this study are available from the author upon request.
